# Middle-aged sandwich generation: the utilization of social capital in coping with the caring demands and threats to mental health

**DOI:** 10.3389/fpubh.2025.1730220

**Published:** 2026-01-13

**Authors:** Gigi Lam, Catherine So-Kum Tang, Tak Sang Chow

**Affiliations:** 1 Department of Sociology, Hong Kong Shue Yan University, North Point, Hong Kong SAR, China; 2 Mrs Dorothy Koo and Dr Ti Hua Koo Center for Interdisciplinary Evidence-based Practice and Research, Hong Kong Shue Yan University, North Point, Hong Kong SAR, China; 3 Department of Counselling and Psychology, Hong Kong Shue Yan University, North Point, Hong Kong SAR, China

**Keywords:** caregiver burnout, sandwich generation, social capital, social capital theory, stress

## Abstract

**Introduction:**

The sandwich generation refers to individuals who simultaneously care for children and parents or grandparents. This study explores how they utilize social capital at various levels to manage their responsibilities.

**Methods:**

Ten participants, aged 35–50, who care for both children and older family members, were interviewed. The data was analyzed thematically and deductively coded based on bonding, bridging, and linking social capital.

**Results:**

The study found that the sandwich generation faces time management issues, physical exhaustion, stress, and caregiver burnout. The findings revealed that significant support comes from parents, in-laws, and maids, while broader support from siblings, peers, and neighborhoods is limited. Religious groups were identified as a key source of emotional and spiritual support. Participants valued pre-nursery and kindergarten services but expressed concerns about the government’s unequal allocation of childcare and long-term care resources. The key role of family over government support reflects traditional Chinese family values and social policies.

**Conclusion:**

The study concludes with recommendations for future research and policy development.

## 1 Introduction

Intergenerational care of family members has emerged as a global and irreversible phenomenon due to changing population structures and increased life expectancy (1). In the US, 24.3% of individuals care for both parents and children (52), while in China and the UK, the figures are 36 and 15%, respectively (2, 53). Hong Kong faces similar challenges, with a shrinking young population (14%) and a growing older population (22.4%) in 2023 (3). Faced with an aging population, the middle-aged group aged 35 or above (42.8%) experiences pressure to care for both generations, often with few siblings to share the burden.

The term “sandwich generation” aptly describes this group of people who must care for both their parents and children (4). Initially introduced by Miller (4), it now includes those caring for parents, in-laws, adult children, and grandchildren in their 50’s or 60’s, or middle-aged individuals caring for multiple generations (5). This paper adopts the latter definition, focusing on the middle-aged sandwich generation aged between 35 and 50.

In Hong Kong, a study found that participants cared for an average of 1.7 children, with 30% also caring for older individuals (6). Another study showed that younger caregivers (aged 34 or below) equally cared for their children (35.9%) and parents (30.8%), while those aged 35 to 54 prioritized caring for children (53.2%) over parents (33.1%) (7).

Apart from the caring responsibilities, sandwich generations are also short of 1.7 sleeping hours and 2.4 leisure hours when compared to Hong Kong citizens in general (7, 8). An international comparison reveals that 46.8% of participants take above 71 h of caregiving, which far exceeded their counterparts in UK (1.3%), US (2.4%) and China (0.8%). Moreover, when asked whether the caregivers feel tired and stressed, the caregivers showed higher score of stress (11.7 out of 16) than their counterparts in UK (9), US (9.8) and China (8.6) (7).

Given the caring responsibilities and stress of sandwich generation in Hong Kong, the current study aims to investigate the social capital utilized by this group in coping with dual caregiving roles or triple job demands.

## 2 Literature review

While the literature on the sandwich generation is extensive, existing research tends to be Western dominated, despite cultural differences (individualism vs. collectivism) and varying welfare systems between Eastern and Western countries (9). One example of the Western research resorts to possible sources affecting the mental and physical health of sandwich generation. The sources include role strain [i.e., an incompatibility of caregivers’ time, energy and resources across competitive demands arising from various roles; (10)], time constraints (11), chronic fatigue (12), low social support (13), negative emotional spillover (14), a feeling of guilt caused by a competition of demands across multiple generations (15, 16) and competitive demands in self-development, career, and finances (17, 18) as postulated by the stress process model.

In addition to the sources affecting the mental and physical health of sandwich generation, the Western-dominated research investigated the deleterious effect on social isolation and mental health, which can be observed in an American sample of dual caregivers of people with dementia (19), a sample of working women (20) and a sample of caregivers from the sample of National Long Term Care Survey (21).

Similarly, the limited Asian studies shared similar tenet with the Western studies. For instance, caregivers of older adults expressed more depressive symptoms and lower scores of life satisfaction than non-caregivers in a large-scale examination of 4,217 Taiwanese (22). Another large-scale study of 8,065 Chinese found that the carers of both grandchildren and parents showed better cognitive health while no effect of cognitive health was found among the carers of parents only (23). The difference is attributed to the inability to reduce depressive symptoms (23). The family pressure and anxiety were also mentioned by another sample of 4–2-2 families with four grandparents, two parents and two children in China (24). Apart from mental health, the expenditure of education on children had been decreased when the adult children were required to support their parents in an examination of 1,477 Chinese (25). The local research on specific carers or the sandwich generation in Hong Kong also centered around their mental and physical health, without considering the role of social capital in alleviating competing demands (7, 26–29).

Social capital is an umbrella term, encompassing various dimensions of social relationships, interactions, and networks. It generally refers to the resources embedded in social structures, including social norms, trust, and reciprocity, which individuals can access and utilize to achieve personal and collective goals. Different scholars, however, have conceptualized social capital in various ways. While Bourdieu (30) elaborated social capital as an individual property in forms of economic, cultural, and symbolic capital stemming from resources derived from one’s network, Coleman (31) emphasized the collective attributes of social capital that facilitates coordination and cooperation. Putnam (32) rather considered social capital as a public good on civic engagement and generalized trust for the sake of common goals and collective benefits, which stands in the middle between Bourdieu’s (30) and Coleman (31)’s conceptualizations.

Given the complex interconnections of social capital across different levels (i.e., individual, social, and regional/national), a more effective approach to consolidating previous scholars’ seminal work alongside the two pillars of network structure and resources within ties can be found in Woolcock’s (33) work, which further corroborates social capital theory. The direct relevance of social capital as a complex and multidimensional concept to present study is to measure social capital at varying levels, including bonding capital as an individual property (i.e., resources provided through strong relationships that exist within homogeneous groups, such as family, relatives, kinship, close friends and colleagues), bridging capital as a property of individuals and the collective (i.e., resources provided through dissimilar and impersonal connections that exist within heterogeneous groups, such as different cultural, religious and ethnic groups) and linking social capital as a public good (i.e., formal organizations, institutions or figures with power and resources help the individuals or communities lacking resources to gain access to information, resources or ideas, such as government and nongovernmental organizations) at different levels, including micro, meso and macro social capital (32–34).

The relevance of social capital to sandwich generation can be found from the following study. Irawaty and Gayatri (16) found that sandwich generation women, whether employed or unemployed, received financial support from husbands but without much social support in childcare and care for older adults, leading to chronic fatigue and a decline in healthy lifestyle choices. Therefore, it shows that the lack of social support can lead to negative consequences, highlighting the importance of social capital in managing dual caregiving roles. Moreover, the sandwich generation with responsibilities toward both younger and older family members increase the likelihood of informal care duties; nevertheless, these familial bonds can also provide valuable social, emotional, and practical assistance during challenging times, thereby safeguarding against various detrimental health effects (1). Therefore, the presence of familial bonds provides valuable support that helps mitigate the negative effects associated with these responsibilities and contributes to better overall health outcomes. One major question of how sandwich generation utilizes social capital to cope with the conflicting demands across generations still leaves unanswered, especially under the Chinese societies in Hong Kong as a confluence of both Eastern and the Western values and a laissez-faire social welfare policy (35).

To address the research gap, the current study aims at investigating whether and how sandwich generation utilizes the types of social capital (i.e., bonding, bridging and linking social capital) at varying levels (i.e., micro, meso and macro levels) to cope with the challenges and alleviate the threats toward mental and physical health. The present study first identifies the types of demands faced by the sandwich generation, which in turn examines the types of social capital utilized by the sandwich generation at different levels.

## 3 Methodology

Purposive sampling was employed to recruit 10 interviewees who fulfill two requirements of (a) age range from 35 to 50 and (b) taking care of both parents/parents-in-laws/ grandparents and children(ren). Advertisements on recruiting subjects were posted on a WhatsApp group of carers for 1 month from April 1 to April 30, 2023. In-depth and structured interviews lasting for 1–1.5 h were conducted in May 2023. Written informed consent was obtained from each interviewee. All interviews were video recorded. The participants who completed the interview received HK$50 supermarket coupons as an expression of gratitude. This study received ethical approval from the Human Research Ethics Committee of the University affiliated with the research team. As the interviews were conducted in Cantonese, all the interview scripts were transcribed first and checked by two researchers. Deductive thematic analysis was then carried out with NVivo 15, and the themes and codes were identified in line with bonding, bridging and linking social capital at micro, meso and macro levels. The codes and themes were further reviewed and discussed by two researchers to ensure the reliability of interpretation.

## 4 Findings

The characteristics of 10 interviewees are presented in Table 1. 70% of interviewees were females whereas the remaining 30% of them were males. The mean age of 10 interviewees was 41.8 with the youngest age of 35 (participants B and F) and the oldest age of 47 (participant I). All of them had full-time jobs and had employed maids, except participants A and J. The number of children ranged from one to three. The youngest child is 1.5 years old and the oldest child is 13 years old. And the participants simultaneously needed to take care of parents/ parents-in-laws/ grandparents financially and emotionally.

**Table 1:** Profile of participants.

Participant	Gender	Age	Job status	Number of children	Summary of caring situation	Maid
A (participant B’s wife)	F	43	No job, and she has been a fulltime mother for half a year	1 son (1.5 years old)	Her parents live apart from her and they are caregivers of grandchildren. She takes care of her parents financially. She was a caregiver for her grandmother. She has a younger brother who lives with her parents.	No
B	M	35	Fulltime job	1 son (1.5 years old)	His parents are both caregivers of grandchildren and being cared. He has 2 brothers.	
C	M	43	Fulltime job	1 son (2 years old)	He is main caregiver of his children and parents and fulltime breadwinner. His mum is both a caregiver and being cared; his father is living at a home for older adults.	Yes
D (participant C’s wife)	F	42	Fulltime job	1 son (2 years old)	She is a fulltime breadwinner; has 3 sisters living apart from her. She needs to take care of her father.	
E	F	46	Fulltime job	1 son (6 years old)	She is the caregiver of 77-year-old father (living apart from participant E and has mental illness), and her mum passed away in September 2022; her father has the broken relationship with her family members in Mainland China, her father does not allow them to become his caregivers.	Yes
F	F	35	Fulltime job	1 daughter (3 years old)	She is the only child and a fulltime working mum raising a 3-year-old daughter; she supports her parents; received few helps from mother-in-law when her daughter was 1 year old.	Yes
G	F	45	Fulltime job	2 children: son (9 years old) and daughter (13 years old)	She needs to support her parents (living apart) with an elder sister living apart; her mum was a caregiver of her children. Her daughter has emotional problems.	Yes
H (participant I’s wife)	F	44	Fulltime job	2 sons (5 and 11 years old)	She lives with I’s father aged 83; her parents and a younger brother live apart from her. Her eldest son has special educational needs-- autism. Her son was confirmed autism since he was 3 years old.	Yes
I	M	47	Fulltime job	2 sons (5 and 11 years old)	He lives with 83-year-old father and his eldest son has special educational needs.	No
J	F	38	No job, fulltime mother (for 5 years)	3 children: sons (2 and 7 years old), daughter (5 years old)	Parents aged 67 (father) and 68 (mother) are living apart from her. The eldest son has suspected special educational needs. His social and emotional problems are periodic, especially before the examination.	

### 4.1 Competitive demands across the caregiving roles between parents, grandparents and child

There are different competitive demands, including physical exhaustion, time management, stress due to lack of relevant knowledge and uncertainty and interpersonal conflict between parents and children. The physical exhaustion was first raised by participant A as follows:

As a full-time mom of a 1.5-year-old son, participant A does not need to take full care of her parents because they are healthy. Instead, “I felt quite demanding because my husband and I needed to go to hospital back and forth for taking care of grandmom before her death. It is very demanding to take care of my energetic baby as well, such as feeding him milk and food.” (participant A, a fulltime mom with a 1.5-year-old son, healthy parents and a grandmother, no maid).

Another working father mentioned the difficulty in allocating time to both parents and child.

“When my father renovated his house, I sometimes figured out how to help my parents, such as making choice on furniture and raw materials. If I do not have a baby boy, I can spend time on planning or buying raw materials with them.” (participant B, a working father with parents and a 1.5-year-old boy).

In addition, another participant who needs to take care of two sons aged 6 and 11, respectively and a co-resident father-in-law aged 83 feels pressurized and stressed.

“My coresident father-in-law usually is very healthy. But he got sick twice with lung and legs. I felt stressed because I was not sure how he would look like in the future, and the doctor could not diagnose his illness. We are unsure how long he needs to stay in hospital and how to take care of him afterwards. I felt very stressed in this regard.

Because the public hospital did not take care of his leg wound very properly and my father-in-law refused to see doctor until 1 day he got a fever, and he even risked cutting the entire leg. I felt stressed out as a carer because I lacked relevant experience. At that time, I could not leave my job post because my boss might call me anytime. Luckily, the hospital is near our home within 1 h reach…My eldest son was also diagnosed autism and learning procrastination.” (participant C, a working mom with two sons (one son with autism) and one co-resident father-in-law aged 83).

Moreover, another working mother feels discomfort because of the interpersonal conflict between her, her father and her son.

“My father temporarily is physical healthy but not good with mental health. He is depressed and paranoid. My father visits doctor but he gives up taking medicine. Because my mother has just passed away, my father lives alone. We have a lot of quarrels. I mostly avoid meeting my father. I try to find my son to accompany my father. Even my son also finds my father very annoying, they have some conflicts. I arrange some gatherings between my son and my father. I feel discomfort but I stand with it…But the gatherings mostly ended in unhappiness.” (participant E, a working mom with a 70-year-old father with mental illness and a son).

### 4.2 The use of social capital to cope with competitive demands

To cope with the conflicting caring demands across multiple generations, one related topic of utmost importance is how carers utilize existing social capital. The following section presents the use of social capital across three different types, including bonding, bridging and linking at micro, meso and macro levels, respectively.

#### 4.2.1 Micro level: bonding social capital

Bonding capital refers to relationships and ties between individuals with a high degree of network closure. The relevant bonding capital includes parents/ parents-in-law, siblings and relatives.

##### 4.2.1.1 Parents/ parents-in-law

The help and support offered by parents in taking care of children largely depends on the health status of parents. Participants B and C first acknowledged the support offered by their healthy parents and parents-in-laws.

“Because participant A (my wife) is the main carer of baby, our parents not only visit our home for taking care of both baby and my wife but also take the baby to their homes for taking care of.” (participant B, a working father and a husband of a participant A who is a fulltime mom).

“We need to start work at 9 a.m. on weekdays, so we leave the house between 7 and 8 a.m. However, the maid does not begin her duties that early. As a result, we rely on my mother to feed the baby. My wife (participant D), my mother, the maid, and I follow a routine schedule, taking shifts to ensure everything is taken care of.” participant C, a working father of a 2-year-old son and a husband of participant D (a working mom).

The important role of parents is not just limited to taking care of grandchildren but also take care of one’s spouse, which was mentioned by participant C again.

“My father lives in a home for older adults, and my mother visits him there regularly. When my father was admitted to the hospital due to COVID-19, my mother also went to the hospital to take care of him after my wife returned home.” (participant C, a working father of a 2-year-old son, his father lives at a home for older adults).

Due to the important role played by parents in taking care of children, the help and support offered by parents would be attenuated once the health of parents deteriorates. Role exit of parents from caregivers to being cared for was mentioned by participants G and J.

“My father always accompanies me when searching for information on Special Educational Needs (SEN) treatments for my eldest son. Additionally, my parents-in-law live nearby, so they can help by taking my child to school. However, as my mother’s health has deteriorated, my parents-in-law now need to spend extra time caring for her. This has significantly reduced the time they can dedicate to taking care of my child.” (participant J, a fulltime mom of 3 children).

When the health of parents deteriorates, it not only reduces the time on taking care of grandchildren but also affects the mental and physical health of sandwich generation. The caregiver burnout referring to chronic and overwhelming emotional, physical and mental exhaustion was mentioned by participant G.

“Initially, my mother took my child to school…However, as her knees deteriorated, she experienced severe pain and could no longer manage this task. This led to emotional problems and sleep disturbances for her, which deeply worried me. Around the same time, my daughter also began experiencing emotional issues at school. The situation became overwhelming, and my stress levels exceeded normal limits without me realizing it. My physical health was affected as well… Thankfully, I have now passed through the toughest period.” (participant G, a working mom with a mother with knee disease and 2 children).

##### 4.2.1.2 Siblings

In contrast to the indispensable and direct support offered by parents, the type and degree of support offered by siblings change to supplementary one.

“I work from Monday to Friday, leaving only weekends and public holidays to take care of the baby. Therefore, I relied on my two elder brothers to help our parents with renovating the house.” (participant B, a working father with parents and a 1.5-year-old son).

“For instance, when my mom had emotional problems, I discussed with my sister, and we helped together.” (participant G, a working mother with parents and two children).

##### 4.2.1.3 Relatives

Similarly, the magnitude and type of support provided by relatives also change to access to information and emotional support, which was mentioned by participants C, F and G.

“I heard from my uncle (my mother’s brother) about one promotion on performing knee changing operation. Because I am very busy at work, I really ignore my mother’s needs.” (participant G, a working mom with two children and mother with degenerative knee expressing self-blame).

“It should be someone known by my uncle (my mother’s brother). And then my mother asked this person. This is the way of locating one suitable home for older adults for my father.” (participant C, a working father with father who needs to live in a home for older adults and one 2-year-old son).

As the only daughter, participant F does not count on relatives (her uncles and aunts) to take care of her daughter because the relatives live far away from her. Rather, the relatives provide emotional support to her mother.

“I think the brothers and sisters of my parents do not take care of my girl. But they may ask my mother if she is okay when she is sick…The relatives live far away from me. And I think the childcare resources are enough.” (participant F, a working mom with a 3-year-old daughter and she is the only daughter at home).

##### 4.2.1.4 Peers

The support provided by peers is also access to information. One participant mentioned the treatment information offered by her ex-secondary school classmate whereas another participant mentioned the medical malpractice information provided by her peers.

“I had not been in touch with this classmate for nearly 20 years when I found her on Facebook. I discovered that she now works at Gleneagles hospital, so I called her immediately. She was very kind and explained the entire knee replacement surgery procedure to me…Thanks to her guidance, my mom was able to have the surgery within two weeks.” (participant G, a working mom with mother having knee problems and two children).

“My mother was admitted to the hospital within 48 h and diagnosed with intestinal perforation, adhesion, and multiple organ failure. Sadly, she passed away. My father was very frustrated and questioned why it happened so quickly. I consulted the doctor to determine if it was medical malpractice and asked my classmates, who said it was probably not.” (participant E, a working mom with dad suffering from mental illness and a son).

Another fulltime mother joined the mother forum for gaining access to resources.

“This is mainly organized by our mother forum. Mother forum mainly mails the resources to mothers during the pregnancy, including diapers, milk powder and supplements.” (participant J, a full-time mother with 3 children).

##### 4.2.1.5 Neighborhood

In contrast, the support provided by neighborhood is very minimal. Only one participant mentioned the brief support provided by neighbors in checking homework for her son with special educational needs (SEN) but it ended in failure.

“Later, we asked for one neighbor’s help with my son’s homework. My eldest son visited the neighbor’s home to do homework. But the neighbor started disliking my son because my son had some strange behavior.” (participant H, a working mom with a coresident father-in-law and two sons, the eldest son has autism).

#### 4.2.2 Micro level: outsourcing housework and caring to domestic helpers

In addition to aforesaid bonding social capital, people can employ domestic helpers to cope with double demands arising from taking care of parents and children. Maids provide additional support, including household chores, taking care of both parents and children and even hygienic issues. Participant G feels very happy and lucky to employ one helpful maid.

“My maid has been working for 13 years. She has been working since the birth of my first child and takes care of my two kids till now.” (participant G, a working mom with mother with knee disease and 2 children).

In addition to taking care of children, maids also offer support to take care of parents.

“But my maid sometimes needs to take care of my mom…My mom fell suddenly; her face hurt and glasses broke. My maid needed to take care of my mom under this circumstance.” (participant C, a working father with mother, father living at home for older adults and one 2-year-old son).

The maids’ duties of taking care of both children and parent are also shared by participants H and I.

“When my father-in-law was admitted to hospital because of COVID-19, my maid brought the meals to my father-in-law. My maid also takes care of my sons.” (participant H, a working mom with a co-resident father-in-law and two sons).

Participant I, the husband of participant H, also mentioned the carer role played by the maid.

“When my father was first discharged from hospital, we learn how to clean his wound on legs. When my father gets better, we pass the job to maid who takes care of his wound.” (participant I, a working father with a co-resident father and two sons).

On the contrary, the absence and inability of visiting Hong Kong by the maid during COVID-19 caused inconvenience and difficult time for participant J.

“I originally had a maid during COVID-19. But the maid left Hong Kong during the pandemic, and we cannot employ maid afterwards. I did not have any maid during the pregnancy of my third child so that it was a very tough time.” (participant J, a fulltime mom with 3 children).

#### 4.2.3 Meso level: bridging capital

##### 4.2.3.1 Religion

Conversely, the support provided by religion is rather comprehensive by covering emotional support, spiritual support, access to information and resources. Emotional and spiritual support were first mentioned by participant E.

“My father joined the church as a protestant two years ago. My father gets mental illness and my mother suddenly passed away. All the people in church are very supportive.” (participant E, a working mom with a dad with mental illness and a son).

“I felt okay about my mother’s death. I prayed to God that there was no medical malpractice, no need to undergo autopsy, no legal procedure and no need to call police…I also hand my dad’s problems to God as a spiritual support.” (continually mentioned by participant E, a working mom with a dad with mental illness and a son).

In addition to emotional and spiritual support, another support provided by religion is access to information.

“Our church has small groups. The members in groups are older than us. My husband and I are the youngest. They are mostly around 50–60 years old. They are experienced in taking care of children and older adults. They are very good at giving us opinions. We go to church during COVID-19 for relieving our pressure.” (participant H, a working mom with a coresident father-in-law and two sons).

Another participant mentioned the access to resources, including educational resources and the other resources.

“Our church has a pre-nursery class, which my son has attended since he was seven months old. As an only child, this class allows him to meet other children and even some older kids. It’s beneficial for his socialization. The pre-nursery class enables my son to play with others and engage in various activities, which is much better than getting bored at home.” (participant D, a working mom with one 2-year-old son).

The access to resources includes not only educational resources but also resources to burial service.

“I passed the burial-related matters of my mom to my church.” (participant E, her mother just passed away and her father gets mental illness).

#### 4.2.4 Macro level: linking social capital

##### 4.2.4.1 Government

The resources provided by government include both childcare services and long-term care services, but the participants had diverse opinions toward the childcare services and long-term care services, especially the services provided to SEN students. On one hand, participants unanimously appreciated the long-term care services provided by the government.

“Because my father went bankrupt, he qualifies for categories covered by Comprehensive Social Security Assistance (CSSA) and fruit money… I spend about HK$1,000 per month on air-conditioning and miscellaneous expenses at the home for older adults. However, the other expenses are covered by government-provided social welfare.” (participant C, a working father with father living at home for older adults and a 2-year-old boy).

Participant A echoed participant C’s idea on long-term care services offered by government.

“Even though my grandmother had not applied for CSSA before, she did not have to pay for medical fees at the hospital or residential fees at the home for older adults. I think the government is very generous and efficient in helping older adults.” (participant A, a fulltime mother with a grandmother and one 1.5-year-old son).

On the other hand, participants expressed divergent views on childcare services provided by the government. One participant agreed that kindergarten can relieve her pressure while another participant expressed dissatisfaction with the other childcare services.

“I believe that full-day kindergarten provides essential social support for working parents like us. Many people do not trust maids to educate children, and our parents or parents-in-law are getting older and may find it demanding to take care of children. Therefore, I chose the full-day kindergarten class for my child to relieve the pressure of childcare.” (participant F, a working mother with one 3-year-old daughter).

“We are familiar with the pre-nursery class, which children can attend from the age of two. It is very beneficial as children can spend almost half of the day there… However, apart from the pre-nursery class, I am not aware of other government services for young children. I believe that support for nursery homes and childcare is insufficient… While more social welfare and resources have been devoted to supporting older adults in recent years, there is inadequate support for people who want to have children or those in the sandwich generation.” (participant A, a fulltime mother with a grandmother and one 1.5-year-old son).

Similar views on the unequal distribution of resources on children and older adults are shared by participant C.

“I am not familiar with how childcare services operate. My colleagues have mentioned that there are limited options available. In contrast, I often hear more about services and subsidies for seniors in the media. Regarding childcare services, it is unclear whether the government or non governmental organizations provide these services. Even if they do, awareness and marketing are not very effective. The current population distribution has a low proportion of babies and a larger proportion of older adults, so it is understandable that the government prioritizes support for older adults.” (participant C, a working father with a 2-year-old son and a father living at the home for older adults).

Apart from unequal distribution on services, participants J and C expressed concerns about childcare services provided by the government.

“Nursery homes are extremely limited, essentially nonexistent. I must rely on myself to take care of my children. If I work, I cannot manage both my job and childcare unless we hire maids or depend on parents for support… During my pregnancy, without maids, the government did not provide any support. The resources are insufficient and mismatched.” (participant J, a full-time mother with 3 children).

“Of course, the aforesaid pre-nursery classes are useful but there is no other service provided by the government. Conversely, I browse through the forum via Facebook and know that someone who can work part-time to take care of children.” (participant C, a working father with a 2-year-old son).

Resource scarcity on SEN students was further mentioned by two parents, one with a son diagnosed as autism by children development center during age 3 and another with a 7-year-old son with autism but still undiagnosed as a SEN student.

“My son has autism and learning delays (autism spectrum), and his muscle development is about a year behind his peers. Under these circumstances, he qualified for the Integrated Program in Kindergarten-cum-Child Care Center (IP), which offers integrated services provided by kindergartens. The kindergarten provided special training for him, such as muscle development exercises. He did not need to go for follow-up sessions. This service was available until he started primary school because he is a SEN student… I think the support provided by the school is sufficient and quite comprehensive now.” (participant H, a working mom with a son with autism).

But participant H’s husband disagreed with her on the support offered to SEN students.

“IP is the resource provided by government. Psychologists and psychiatrists are the treatment resources. There is nothing else. We need to figure out the other ways on academics and other development.” (participant I, a working father with a son with SEN).

In contrast to treatment mentioned by participants H and I, the assessment and diagnosis were mentioned by participant J who has a 7-year-old son with autism but still undiagnosed as a SEN student.

“The evaluation on SEN made by private sector is very expensive…Or we wait for government, but the waiting time is very long…I basically cannot utilize any resources provided by government because we did not bring our son for assessment.” (participant J, a fulltime mom with one 7-year-old boy with autism but still undiagnosed).

##### 4.2.4.2 Nongovernmental organizations

In addition to the resources and services provided by the government, the participants also utilized the relevant resources provided by non governmental organizations.

“YMCA is near my home, and it provides services for the teenagers and children. At that time, I always saw one thing called emotional management and then I consulted the social worker. I told the social worker I felt helpless because I did not know how to help my girl…Then we enrolled in eight 1-to-1 lessons.” (participant G, a working mom with 2 children and the eldest girl has emotional problems).

The utilization of existing resources offered by nongovernmental organizations is also shared by another participant.

“Perhaps there is one Playground Association with some playground facilities in garden and library with staff. I used these playground facilities before. They are all free.” (participant F, a working mom with a 3-year-old daughter).

Differing from the positive experience shared by participants F and G, the couple (participants I and H) shared a bad experience of using services provided by nongovernmental organization.

“We have sought help from one nongovernment organization. We participated in game therapy, which is for the SEN students…After a few months, we found that our son did not feel happy and said he always lost the game. Therefore, we observed and felt that children with my son in the same class were the normal ones.” (participant I, a working father with a son with SEN).

Participant H continually explained and concluded this practice as a misuse of resources.

“In fact, these activities are challenging to wait for. I believe they fail to provide adequate services to those truly in need because the services are not based on clinical reports as screening criteria, but rather on the decisions made by occupational therapists… Additionally, the NGO places both suspected SEN students and confirmed SEN students in the same class, leading to a misuse of resources.” (participant H, a working mom with a son with SEN).

## 5 Discussion

The sandwich generation expressed difficulties in time management, physical exhaustion, and mental health problems such as stress and caregiver burnout. Participant H also mentioned the conflict between her job, caring for her sons, and her father-in-law, referring to triple-duty caregivers. This aligns with the negative psychological outcomes faced by the sandwich generation (36) and the challenges encountered by triple-duty caregivers (37).

To cope with the challenges from dual caregiving roles, the sandwich generation adopted problem-focused coping by utilizing bonding, bridging, and linking social capital at micro, meso and macro levels. They tended to rely heavily on support from their parents, parents-in-law, and maids, while the support from other domains, including siblings, relatives, peers, and neighbors, decreased in magnitude and importance within bonding social capital. This reliance on extended family members for childcare and outsourcing housework and family care to domestic helpers was evident (38).

In contrast, religion as a bridging social capital can offer emotional, resourceful, and spiritual support to the sandwich generation. This can be seen as interactive social capital that helps form, maintain, and end relationships (39). The last type, linking social capital, is a combination of government and nongovernmental organizations. While participants appreciated pre-nursery classes and kindergartens, they still had reservations about the adequacy and awareness of childcare services and the disproportionate long-term care services allocated to older adults. It is in line with the increasing but limited child services before age 3 and SEN services discussed by Xia and Ma (40) and the actual expenditure allocated to family and child welfare (4.5%) and long-term care services (14%) by the Social Welfare Department (41).

In addition to the social capital within each level, a close linkage can be identified across three different levels. For instance, when participant G felt inadequacy of bonding social capital in taking care of her daughter with emotional problems and her mother with degenerative knees, she would identify the other types of social capital, such as discussing with her sister, obtaining relevant information from her uncle, asking her ex-secondary school classmate via Facebook, and seeking help from nongovernmental organizations. Similarly, when participant C felt insufficiency, he supplemented the inadequacy with support provided by the religious group, browsing through Facebook forum and the corresponding long-term care and childcare provided by government.

The findings indicate that members of the sandwich generation tend to mobilize broader, more diverse, and multi-level forms of social capital to manage their dual caregiving responsibilities. This pattern contrasts sharply with the more specialized and life-stage-specific social capital utilized by single-focus carers, such as those caring exclusively for parents or children. Specifically, carers for parents predominantly rely on bonding social capital, such as close family ties, and linking social capital, which connects them to formal institutions like healthcare systems and centers for older adults (42, 43). In contrast, carers for children primarily draw on bonding social capital through parental networks for emotional reassurance and advice, as well as bridging social capital through school communities, playgroups, and online parenting forums (31, 44). These distinctions underscore how caregiving roles shape the structure and function of social networks, with sandwich carers requiring cross-domain connections to navigate complex, overlapping responsibilities.

In addition to the distinctiveness between sandwich generation and the other types of carers, the findings also offer credence to social support theory in terms of reliance on parents, parents-in-law, and maids, with minimal government support, resulting from micro–macro interactions. Despite financial uncertainties and challenges like unemployment, lack of pensions, low fertility, and high divorce rates (45, 46), our findings showed that the sandwich generation still provides instrumental, financial, and emotional support to parents in daily routines and health-related issues. Additionally, parents often help the sandwich generation by caring for grandchildren, depending on their age, health, and willingness, which acts as bonding social capital. While some argue that filial piety is fading (54), a study of 505 Hongkongers showed that most viewed caregiving as an adult child’s responsibility, even without social pressure (55). Caregiving, however, depends on parents’ health, living arrangements, finances, and relationships (55).

Intergenerational transfers, from parents to children, then adult children to parents, and grandparents to grandchildren, reflect reciprocal family relationships. This generalized reciprocity involves altruistic caregivers who do not expect immediate or equivalent returns (56, 57). Long-term parent–child relationships are based on the understanding that adult children will care for parents in return for early parental investment (58). Parents also support adult children through financial aid, housework, or babysitting, reciprocating the care they receive (59). This dynamic may shift with parents-in-law, where equity is prioritized (60). Support for grandchildren may lead to later support for parents-in-law, while lack of initial support may reduce the obligation to care for them (60). For example, participant H cares for her father-in-law because she initially received childcare support from him.

At a macro level, the residual welfare system, strengthened by positive noninterventionism and economic pragmatism, prioritizes economic success over welfare provision (40, 47). This system involves the government relinquishing its primary responsibility for social welfare to families, advocating traditional Chinese welfare ideologies such as filial piety, humanity, and diligence, as discussed by the first Chief Executive, Tung Chee-wah (48). The philosophy of social policy was reinforced by the *White Paper on Social Welfare into the 1990s and Beyond* (49), which states, “The primary responsibility for the adequate care of children rests with parents, and the separation of children from their families should be tolerated only where there is no better alternative.”

This interaction between family values and government policy positions Hong Kong as a unique hybrid within Esping-Andersen’s (50) typologies of welfare regimes, characterized by low decommodification (the effectiveness of state policy in reducing dependence on the labor market for welfare needs) and low defamilialization [the effectiveness of state welfare policy in reducing familial dependence; (46)]. This productivist welfare regime subordinates social policy to economic growth and familistic traditions, supported by low to middle tax rates (51). Similar distinctions are faced by other Asian countries, such as South Korea, Japan, and Singapore, in differentiating between Esping-Andersen’s typologies (46).

To address these issues, childcare, long-term care, and population policies should be integrated. Enhancing awareness and ensuring adequate childcare and SEN services are crucial. Collaboration between the government, NGOs, and mother forums can improve support. Training sessions for older adults on grandchild care should be continued to promote productive aging. Economic incentives, such as tax concessions, could support families employing domestic helpers. The well-being of older adults must be considered, as they may become part of the sandwich generation themselves. This aligns with a study by the Hong Kong Council of Social Service (7), which found that caregiving responsibilities shifted to spouses (42.9%), children (26%), and parents (22.8%) when caregivers are over 55.

## 6 Conclusion

The sandwich generation face dual or sometimes triple demands from caring for parents, children, and managing a job, leading to caregiver burnout and self-blame. Problem-focused members of the sandwich generation utilize social capital effectively at micro, meso, and macro levels. While parents, parents-in-law, and maids provide significant support, the assistance from siblings, relatives, peers, and neighbors varies and diminishes in importance. Religious groups at the meso level offer substantial emotional and spiritual support, functioning as interactive social capital that helps form, maintain, and end relationships (39). At the macro level, linking social capital combines government and non governmental organizations. Participants unanimously praised the efficiency of pre-nursery classes and kindergartens but expressed concerns about the awareness and adequacy of childcare services, echoing findings from a consultative study by the University of Hong Kong (51). This reflects the interplay between cherished family values and government social policy direction (46).

As an exploratory study on social capital utilized by the sandwich generation, this research has several limitations. Firstly, it targeted 10 middle-aged individuals without comparison groups. It is limited by a small sample size. Moreover, the sandwich generation also includes older adults who care for both spouses and grandchildren. Future studies should involve diverse comparison groups with a large sample size, such as individuals with varying socio-economic statuses and age groups. Secondly, this study is cross-sectional, failing to capture the dynamics and life transitions faced by the sandwich generation. It relies on participants’ memories, such as participant H, who has children aged 6 and 13. Future research should adopt a longitudinal approach. Lastly, this qualitative study uncovered in-depth personal experiences and opinions but is limited by a small sample size. Future research should employ a mixed-methods approach, starting with quantitative research to assess participants’ mental health using psychological instruments, followed by qualitative research.

## Data Availability

The raw data supporting the conclusions of this article will be made available by the authors, without undue reservation.
